# Mosquito antiviral defense mechanisms: a delicate balance between innate immunity and persistent viral infection

**DOI:** 10.1186/s13071-019-3433-8

**Published:** 2019-04-11

**Authors:** Wai-Suet Lee, Julie A. Webster, Eugene T. Madzokere, Eloise B. Stephenson, Lara J. Herrero

**Affiliations:** 1 Institute for Glycomics, Griffith University, Gold Coast Campus, Southport, QLD 4215 Australia; 2 Environmental Futures Research Institute, Griffith University, Gold Coast Campus, Southport, QLD 4215 Australia

**Keywords:** Mosquito, Antiviral defense, RNAi pathway, Persistent infection, Transmission-blocking strategies

## Abstract

Mosquito-borne diseases are associated with major global health burdens. *Aedes* spp. and *Culex* spp. are primarily responsible for the transmission of the most medically important mosquito-borne viruses, including dengue virus, West Nile virus and Zika virus. Despite the burden of these pathogens on human populations, the interactions between viruses and their mosquito hosts remain enigmatic. Viruses enter the midgut of a mosquito following the mosquito’s ingestion of a viremic blood meal. During infection, virus recognition by the mosquito host triggers their antiviral defense mechanism. Of these host defenses, activation of the RNAi pathway is the main antiviral mechanism, leading to the degradation of viral RNA, thereby inhibiting viral replication and promoting viral clearance. However, whilst antiviral host defense mechanisms limit viral replication, the mosquito immune system is unable to effectively clear the virus. As such, these viruses can establish persistent infection with little or no fitness cost to the mosquito vector, ensuring life-long transmission to humans. Understanding of the mosquito innate immune response enables the discovery of novel antivectorial strategies to block human transmission. This review provides an updated and concise summary of recent studies on mosquito antiviral immune responses, which is a key determinant for successful virus transmission. In addition, we will also discuss the factors that may contribute to persistent infection in mosquito hosts. Finally, we will discuss current mosquito transmission-blocking strategies that utilize genetically modified mosquitoes and *Wolbachia-*infected mosquitoes for resistance to pathogens.

## Background

Mosquito-borne viruses are a global health priority due to frequent resurgences of major epidemics and unprecedented geographical expansion in the last few decades [1]. The majority of mosquito-borne viruses are RNA viruses, with either single or double-stranded RNA that have positive or negative polarity. Viruses primarily associated with global morbidity and mortality are from the following families: *Flaviviridae* (genus *Flavivirus*, positive single-stranded RNA), *Togaviridae* (genus *Alphavirus*, positive single-stranded RNA) and *Bunyaviridae* (genus *Phlebovirus*, negative single-stranded RNA) [2, 3]. Viruses from the *Flaviviridae* family include yellow fever virus (YFV), dengue virus (DENV), Japanese encephalitis virus (JEV), West Nile virus (WNV) and Zika virus (ZIKV) [4]. DENV is considered the most important mosquito-borne virus, causing 390 million dengue infections annually, and is primarily transmitted by *Aedes aegypti* [5]. Dengue epidemics have expanded significantly in the last few decades to at least 128 countries [6]. *Aedes aegypti* is also the primary vector for other flaviviruses, including YFV and ZIKV. Zika virus infections are typically mild or asymptomatic, but have been linked to Guillain-Barré syndromes in adults. Zika virus infections are also a major concern to pregnant women and are associated with birth defects, such as microcephaly in prenatally infected infants [7]. In February 2016, ZIKV epidemics were declared a public health emergency by the World Health Organization (WHO), with 62 countries and territories reporting evidence of transmission since its introduction into Brazil in 2015 [8, 9]. Some flaviviruses, including JEV and WNV, are primarily transmitted by mosquitoes belonging to the genus *Culex* [8, 10]. Members that belong to the *Togaviridae* family include chikungunya virus (CHIKV), Sindbis virus (SINV), Semliki Forest virus (SFV) and Ross River virus (RRV) [11]. The *Anopheles* mosquito, which is the main vector for the parasite *Plasmodium falciparum*, is only known to transmit O’nyong-nyong virus (ONNV), which belongs to genus *Alphavirus* [12].

Following ingestion of a viremic blood meal from an infected vertebrate host, the virus initiates infection in the mosquito midgut. However, the dissemination of virus from the midgut to salivary glands is not well understood. It is postulated that once the virus enters midgut epithelial cells, replication occurs in the cells and the virus subsequently spreads to the hemocoel [13]. The hemocoel is an open body cavity where hemolymph circulates, and thus once the hemolymph is inoculated, the virus can spread to other secondary tissues *via* hemolymph circulation, including the salivary gland, fat body, trachea, muscles and neural tissue [14, 15]. The spread of virus to the salivary glands is essential for the mosquito to be competent for virus transmission to subsequent vertebrate hosts [16, 17].

## The mosquito innate immune pathways in mosquitoes

The mosquito innate immune response is a key determinant for successful transmission of viruses. Unlike the mammalian immune system, mosquitoes do not possess adaptive immune responses and are dependent on innate immunity to fight viral infection. Most of the knowledge on insect antiviral innate immunity is elucidated from studies of the genetic model insect *Drosophila melanogaster* [18, 19]. Viral infection triggers the activation of innate immunity pathways and leads to the transcription of genes responsible for antiviral responses.

The innate immune system of mosquitoes consists of two tightly interconnected responses: the cellular and humoral defense responses. These two responses act together to protect mosquitoes against a wide variety of pathogens, including bacteria, yeast and viruses. The cellular defense response includes phagocytosis, nodulation and encapsulation of pathogens by hemocytes [20, 21]. Humoral responses refer to the activation of downstream signaling and effector responses, leading to the synthesis and secretion of soluble effector molecules, such as antimicrobial peptides (AMPs), reactive oxygen species (ROS) and components of the phenoloxidase cascade [14, 22–25]. Downstream signaling and humoral effector responses will be discussed later in this review. These effector molecules are secreted into the hemolymph to control infection caused by invading pathogens [26, 27]. Epithelial cells in the mosquito midgut are the first line of defense against many pathogens which are acquired from blood-feeding and these cells can synthesize several AMPs and ROS. Additionally, the fat body of the mosquito is the primary site of the humoral response *via* production and secretion of AMPs. The transcription of innate immunity genes encoding for AMPs is highly dependent on several signaling cascade pathways, including the Janus kinase-signal transducer and activator of transcription (JAK-STAT), Toll and immune deficiency (Imd) pathways [9, 14, 25, 28, 29]. Although activation of these pathways has been shown to limit viral replication, the most robust antiviral defense is the RNA interference (RNAi) pathway. The RNAi pathway produces small RNAs using viral double-stranded RNA as a template to ultimately target the viral RNA for degradation, thereby inhibiting viral replication [30]. Virus recognition is mediated by pattern recognition receptors (PRRs) that recognize virus-conserved pathogen-associated molecular patterns (PAMPs) to initiate innate immune responses.

### RNA interference (RNAi) pathways

**Fig. 1:**
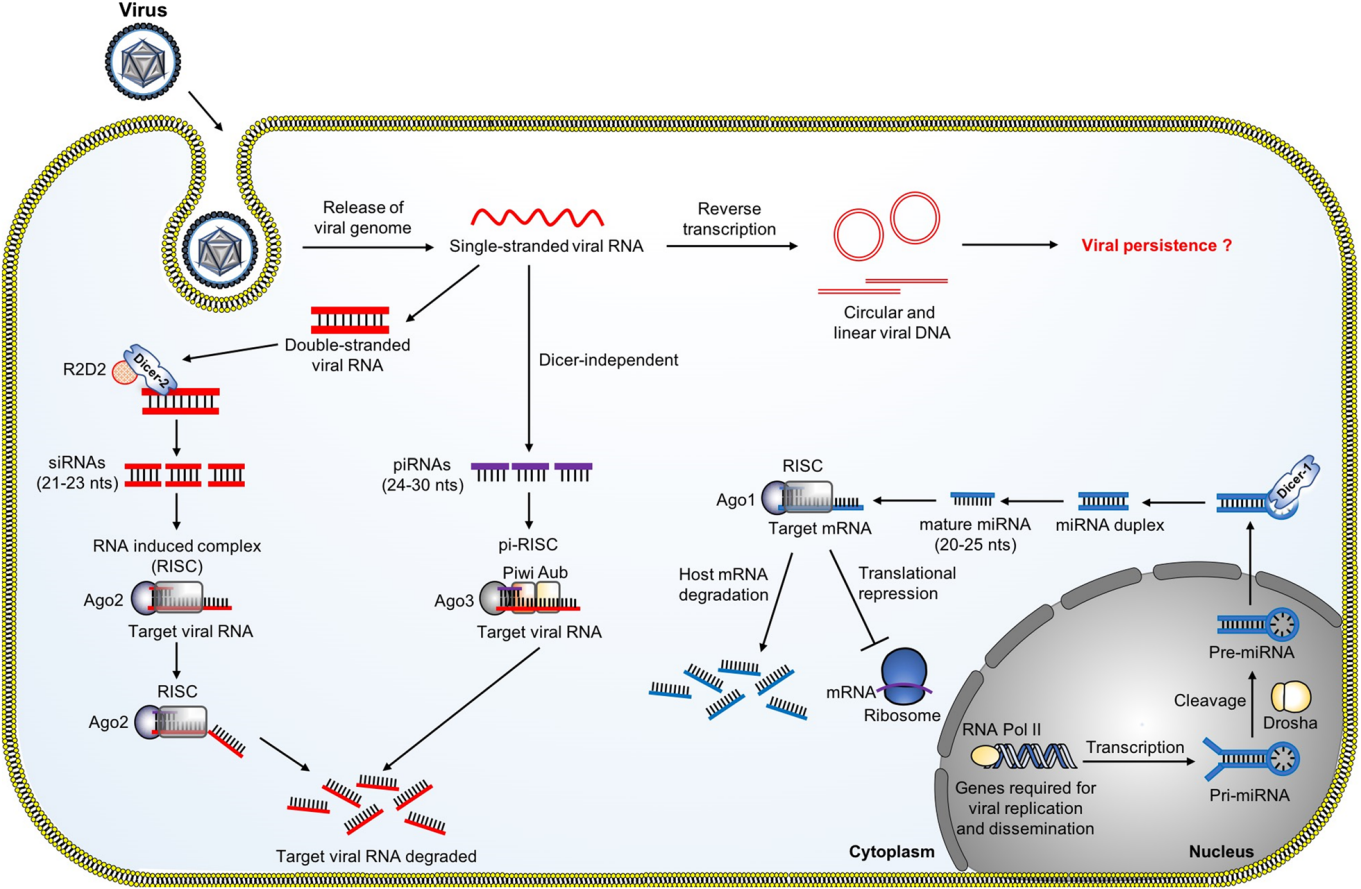
The RNAi pathways in mosquitoes. The three major types of small RNAs present in mosquitoes are small interfering RNAs (siRNAs), microRNAs (miRNAs) and Piwi-interacting RNAs (piRNAs), with siRNAs being the main antiviral response in mosquitoes

RNA interference (RNAi) is the central antiviral mechanism in insects, particularly in controlling virus infection through degradation of RNA, also known as RNA silencing. The key event in the RNAi pathway is the production of small RNAs from long viral double-stranded RNA (dsRNA) (Fig. 1) [31]. There are three major types of small RNAs: (i) small interfering RNAs (siRNAs), (ii) microRNAs (miRNAs), and (iii) PIWI-interacting RNAs (piRNAs), with siRNAs being the main antiviral responses in mosquito. Mosquito-borne viruses are primarily RNA viruses, with their genomes comprised of single-stranded RNA that is either positive-sense (+) or negative-sense (−) [30, 32]. During genome replication, these viruses generate dsRNA as replication intermediates [33]. The siRNA pathway is responsible for the major antiviral response [34]. The viral dsRNA binds to a Dicer-2-R2D2 complex, which consists of a RNase III enzyme, called Dicer-2 (Dcr-2), and an associated protein, called R2D2 [35]. The dsRNA is cleaved by the RNase III domain of Dcr-2 to produce siRNAs of 21–23 nucleotides (nt) in length [36]. The siRNAs then activate the RNAi machinery by binding to another multiprotein known as the RNA-induced silencing complex (RISC), in which one of the RNA strands is degraded. The remaining single-stranded RNA (ssRNA) then serves as a guide-strand to detect and degrade cognate viral RNA by host endonuclease, Argonaute-2 (Ago2) in a sequence specific manner [31]. One study has demonstrated that silencing of the RNAi pathway during DENV2 infection in *Ae. aegypti* enhanced virus replication, indicating their role in controlling viral replication [37]. Transgenic *Ae. aegypti* mosquitoes with RNAi pathway impairment in the midgut were observed to have enhanced SINV replication in the midgut and increased virus dissemination rates [38]. In DENV-infected *Ae. aegypti*, virus-specific siRNAs (20–23 nt), piRNAs (24–30 nt) and unusually small RNAs (13–19 nt) were detected [39]. The siRNA pathway is also an elicited antiviral response in *An. gambiae* against ONNV infection [40].

miRNAs are a class of endogenous small non-coding RNAs (20–25 nt) and play significant roles in the post-transcriptional regulation of target genes in multiple metabolic processes by cleavage of target mRNAs or repression of mRNA translation [41, 42]. Similar to the siRNA pathway, the miRNA pathway starts with cleavage of the dsRNA into small dsRNA, which is loaded into the RISC and serves as a guide-strand to detect and degrade cognate viral ssRNA. The differences between the siRNA and miRNA pathways are the cellular compartments and the effector proteins involved in the pathways [43]. The transcription, cleavage and processing of siRNA mainly take place in the cytoplasm while the miRNA genes are transcribed into primary miRNA (pri-miRNA) by host polymerase II and are processed into precursor miRNA (pre-miRNA) by Drosha in the nucleus. The pre-miRNA is then exported into the cytoplasm and further processed into a mature miRNA by Dicer-1 and is loaded into Ago-1 of the RISC, which guides the binding of the complex to complementary mRNA for degradation [44]. The antiviral role of miRNAs in mosquitoes has not been reported as it was assumed that RNA viruses do not generate miRNAs. This is because of a lack of access to the Drosha for miRNA processing in the nucleus as replication of most RNA viruses occurs in the cytoplasm [45, 46]. However, miRNAs from a number of arbovirus mosquito vectors have been shown to play a critical role in modulating host genes to control viral infection. For example, several miRNAs specific for innate immunity and multiple metabolic processes required for viral replication and dissemination were modulated during ZIKV [47], DENV [48] WNV [49] and ONNV infections [50].

Besides the well-studied siRNA pathway, recent studies have highlighted the importance of the piRNA pathway in the mosquito antiviral response [51–53]. Interestingly, the piRNA pathway can mount an antiviral defense with a defective siRNA pathway, indicating the redundancy of RNAi-mediated antiviral immune responses [51]. In contrast to siRNAs, the biogenesis of piRNAs does not require Dicer and the size distribution of piRNAs is around 24–30 nt [54]. In *Drosophila*, the biogenesis of piRNAs involves three Piwi proteins, including the P-element induced wimpy testis (Piwi), Aubergine (Aub) and Argonaute 3 (Ago3), to form a piRNA-induced silencing complex (piRISC) [55]. The biogenesis starts with the primary processing pathway, which is the synthesis of primary piRNA pool from single-stranded precursors. The primary piRNAs can be associated with the Aub and Piwi protein. Interestingly, the primary pool of piRNAs can undergo an amplifying process known as the ‘ping-pong’ cycle to further amplify the Aub-bound piRNAs to refine the piRNA pool. This amplification process serves to ensure an efficient piRNA-mediated silencing of target RNA [52–54]. The presence of virus-specific piRNAs was detected in *Ae. aegypti* and *Ae. albopictus* during CHIKV infection [56] and in SINV infected *Aedes* cells [53]. Deep sequencing data reported the presence of SFV-derived piRNAs and silencing of PIWI 4 protein resulted in increased SFV replication and virion production, suggesting the importance of the piRNA pathway in antiviral immunity [57].

### Viral DNA produced during replication is important for mosquito survival and persistent infection

The mosquito immune response is implicated in virus persistence [30, 58, 59]. Despite activation of mosquito antiviral immune responses during viral infections, viruses are not completely eliminated from the mosquitoes. Instead, a persistent infection, with little or no cost of fitness to the host, is established in mosquitoes, which makes them efficient vectors for viral diseases. However, the mechanisms by which viruses maintain persistent infection in mosquitoes are poorly understood. Recent studies have demonstrated that virus-derived DNA (vDNA) generated during viral infection are important for mosquito survival and persistent infection [30, 58]. The majority of mosquito-borne viruses are RNA viruses, and upon infection, viral RNA or truncated forms of the viral genome produced during virus replication, also known as the defective viral genomes (DVGs), are reverse transcribed to vDNA by the activity of host cellular reverse transcriptase [58]. Although the biogenesis and regulation of vDNAs in mosquitoes has not been well studied, it has been reported that Dcr-2 regulates the production of vDNA from DVGs as illustrated in Fig. 1. Dcr-2 is a multifunctional protein and vDNA production is regulated by its DExD/H helicase domain [58]. vDNAs can be detected not only in mosquito cell culture during infection, but also in *Ae. aegypti* and *Ae. albopictus* during CHIKV and ZIKV infections [30, 59]. The vDNAs then stimulate the RNAi machinery to control viral replication. Interestingly, vDNA is sufficient to produce siRNAs to elicit antiviral response when challenged with a cognate virus. Furthermore, inhibition of vDNA production results in extreme susceptibility to viral infections [30].

### Other innate immune pathways: JAK-STAT, Toll and Imd pathways

**Fig. 2:**
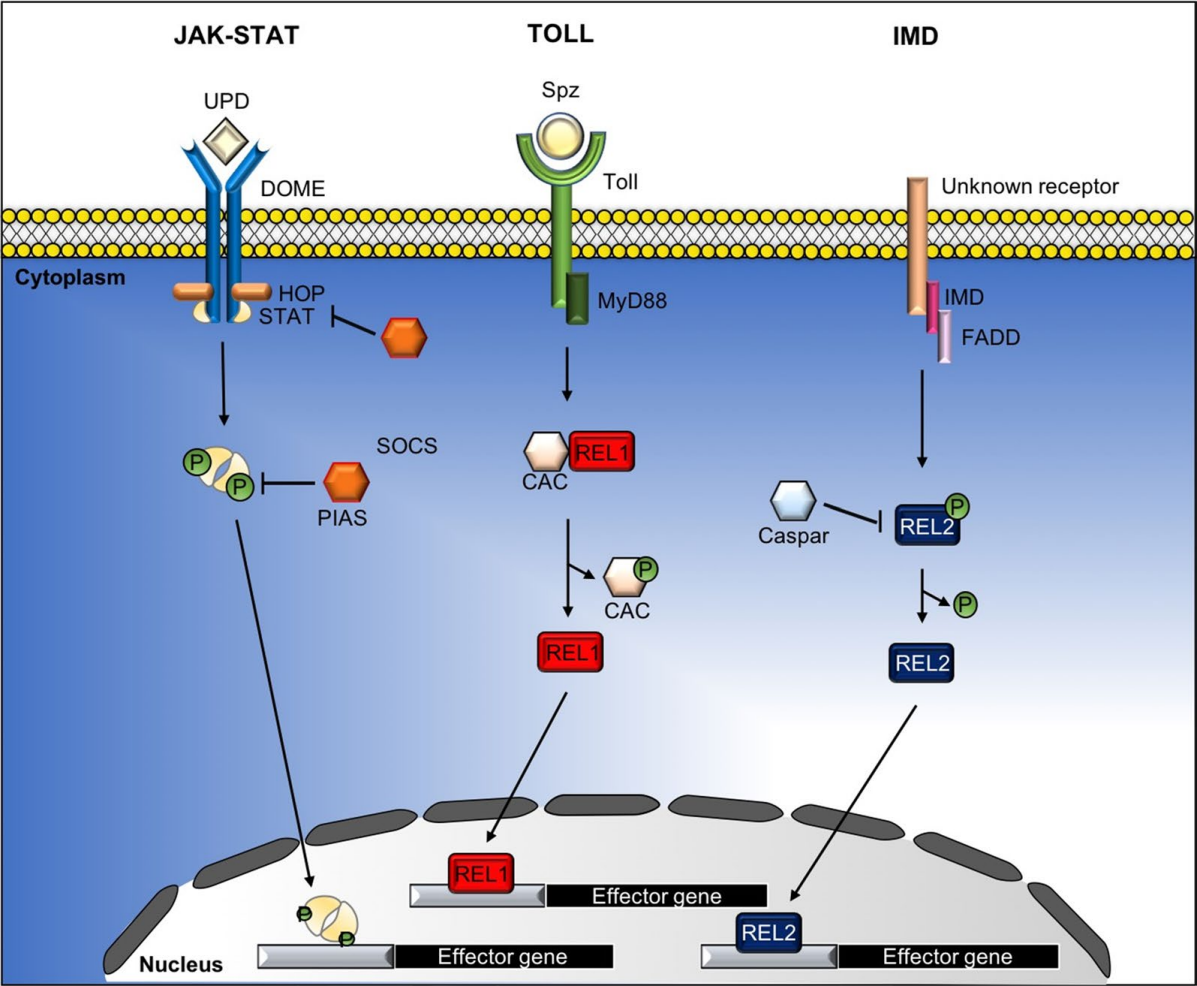
The JAK-STAT, Toll and immune deficiency (Imd) pathways in mosquitoes. Activation of the JAK-STAT, Toll and Imd pathways initiates the formation of a multiprotein complexes consisting of protein kinases, transcription factors and other regulatory molecules to regulate the expression of downstream innate immunity genes, such as the genes that encode for AMPs and key factors that regulate the innate immune system

In addition to the RNAi pathway, there are other innate immune pathways involved in protecting mosquitoes against viral infection, including the JAK-STAT, Toll and Imd pathways (Fig. 2). In response to viral infection, activation of these pathways initiates the formation of a multiprotein complex consisting of protein kinases, transcription factors and other regulatory molecules to regulate the expression of downstream innate immunity genes [14, 22, 60]. These include genes that encode for AMPs and key factors that regulate the innate immune response to viruses. AMPs are immune-inducible peptides that are potent and rapid-acting immune effectors with antimicrobial activities [61]. A wide spectrum of AMPs have been reported in insects during infection with Gram-negative and Gram-positive bacteria, filamentous fungi and yeast [19, 61]. These AMPs carry out both direct killing and innate immune modulation (recruitment and activation of immune cells) to limit invading pathogens [19, 62, 63]. Most studies on the regulation of AMPs during infection are based on *Drosophila*, and the regulation of AMPs in mosquitoes is poorly understood. AMPs vary among different mosquito species and the induction of AMPs is regulated by multiple immune signaling pathways and is highly dependent on the type of pathogen that elicited the response. In *Ae. aegypti*, 17 AMPs have been identified, and they belong to five different families: defensins (cysteine-rich peptides), cecropins (α-helical peptides), diptericin (glycine-rich peptides), attacin (glycine-rich peptides) and gambicin (cysteine-rich peptides) [61, 63, 64]. The mode of killing of AMPs is often specific for different microorganisms. Defensins are active and highly toxic against Gram-positive bacteria and parasites by disrupting the membrane permeability barrier, thereby causing loss of motility [65]. As for cecropins, these positively charged peptides bind to the lipids in the membrane that are negatively charged, thus changing the biological structure of membranes. Other possible modes of killing by cecropins include inhibition of nucleic acid and protein synthesis and inhibition of enzymatic activity [66]. Defensins and cecropins have been found to be expressed in the midgut, thorax and abdominal tissues of *An. gambiae* mosquitoes and are induced during infection with parasite [67]. In the same mosquito species, gambicin has been found to be induced by parasites in the midgut, fat body and hemocytes [64]. However, their role in regulating antiviral immune response is not completely understood. In *Ae. aegypti*, cecropins are upregulated in DENV-2 infected mosquitoes [66]. Furthermore, cecropins exhibit antiviral activity against DENV and CHIKV [66]. In *Culex* mosquitoes, Vago is a secreted peptide regulated by the JAK-STAT pathway and overexpression of Vago reduces the viral load of WNV in mosquitoes [28]. During SINV infection in *Drosophila*, two AMPs, regulated by the Imd and the JAK-STAT pathways, namely the attacin C and diptericin B, control viral RNA synthesis and knocking down of these genes increases viral load in flies [10].

### The JAK-STAT pathway

The JAK-STAT pathway was originally identified in *Drosophila* and was shown to have an active role in antiviral defense against *Drosophila* C virus (DCV) and Flock House virus (FHV) [18]. Consistent with *Drosophila*, mosquitoes also express the cytokine receptor, Domeless (Dome) and the tyrosine kinase Hopscotch (Hop), which together induce the JAK-STAT pathway. The mechanism of the Dome receptor is similar to the mammalian JAK-STAT pathway. The ligand binds to Dome, which then undergoes a conformational shift leading to self-phosphorylation of Hop (JAK). Activated Hop phosphorylates Dome, which forms a docking site for cytosolic STATs. Recruitment of STATs by the Dome/Hop complex leads to the phosphorylation and release of the STATs. The phosphorylated STATs dimerize and translocate to the nucleus where they activate transcription of specific effector genes, such as the virus-induced RNA 1 (*vir-1*) gene, that has a role in antiviral immunity [18, 22]. Through reverse genetic approaches and functional studies, the JAK-STAT pathway has been shown to mediate increased resistance to DENV and ZIKV in infected *Ae. aegypti* [22, 25]. Genetically modifying *Ae. aegypti* to overexpress Dome and Hop renders the mosquitoes more resistant to DENV infection, but not to CHIKV and ZIKV infections. These studies suggest that *Ae. aegypti* possess varied molecular responses to different viruses [68].

The Dome receptor is the most well characterized cytokine receptor in mosquitoes; however, evidence suggests that other cytokine receptors are present which also activate the JAK-STAT pathway. For example, in *Culex* mosquitoes, a secreted peptide known as Vago was upregulated in response to WNV infection, subsequently activating the JAK-STAT pathway to control infection and reduce viral load [28]. However, knockdown of *Dome* did not inhibit signaling of the JAK-STAT pathway, indicating that Vago activated JAK-STAT *via* another unknown receptor [28].

Transgenic *Ae. aegypti* mosquitoes have been used to investigate the role of the JAK-STAT pathway in viral infection. Through RNAi-mediated gene silencing of the tyrosine kinase complex, Dome and Hop increased DENV infection, whereas knockdown of PIAS, a known negative regulator of the JAK-STAT pathway, decreased DENV infection [22]. However, although the JAK-STAT pathway is increased in response to DENV infection in the mosquito, strains that were either resistant or susceptible to DENV infection did not show a difference in viral infection, indicating that the pathway was not involved in viral susceptibility to DENV [69].

The majority of investigations into the JAK-STAT pathway in mosquito immunity have involved dengue infection; however, pathway activation in response to other viruses and downstream mechanisms may differ for each virus. Transgenic overexpression of Hop in the midgut decreased DENV2 infection and dissemination; however, for ZIKV, dissemination was only decreased at day 7 post-infection and infection was not altered [68]. In contrast to ZIKV and DENV, the JAK-STAT pathway was not activated by CHIKV infection [70], nor was it involved in viral dissemination [68]. Furthermore, in human host cells, CHIKV non-structural protein 2 has been shown to inhibit interferon signaling *via* inactivation of the JAK-STAT pathway [71]; however, the precise mechanism of action has not been elucidated. Together, this raises the possibility that the CHIKV inhibitory mechanism acts directly on the JAK-STAT pathway and hence may be conserved in the mosquito immune system. Just as CHIKV may inhibit the JAK-STAT pathway, SFV has also been shown to downregulate transcription of the JAK-STAT pathway [9]. Thus, both CHIKV and SFV have developed mechanisms to avoid activation of this pathway and the downstream effectors of the JAK-STAT pathway are differentially affected between the viruses.

### The NF-κB-like signaling pathways: Toll and Imd pathways

The Toll and Imd pathways are two distinct innate immune pathways very similar to the mammalian NF-κB signaling pathway, which is the key regulator in the production of AMPs. The Toll pathway was first reported in *Drosophila*, and is known for its role in innate immunity against pathogens, such as fungi and Gram-positive bacteria [72]. In contrast, the Imd pathway is activated during infection by Gram-negative bacteria [72]. Both Toll and Imd pathways are activated by pathogens *via* binding of PAMPs to the host’s PRRs, which leads to a cascade of events to activate immune effector genes for production of AMPs. The Toll pathway is initiated by cleavage of the cytokine Spätzle (Spz), which is a ligand that binds to the Toll transmembrane receptor. Activated Toll triggers signaling through MyD88, Tube (adaptor proteins associated with Toll) and the Pelle kinase. Subsequently, the negative regulator of the Toll pathway, Cactus, is phosphorylated and undergoes proteasomal degradation that cause the translocation of the transcription factor Relish 1 (Rel1) from the cytoplasm to the nucleus and binding to κB motifs on the promoters of many AMPs genes, such as *Diptericin* and *Cecropin* that are active against fungi and Gram-positive bacteria [73]. While in the Imd pathway, activation of the pathway leads to degradation of the negative regulator Caspar, which leads to the translocation of Relish 2 (Rel2) to the nucleus, resulting in the transcription of AMPs [14, 74].

The majority of studies on the Toll and the Imd pathways are focused mainly on their antifungal and antibacterial functions in mosquitoes [73]; however, their role in antiviral immune response is not well characterized. Comparative genomic analysis between *Drosophila* and mosquitoes revealed that the key components of the Toll and the Imd pathways are conserved between these two species. The homologues of genes from the Toll and the Imd pathways can be found in *Ae. aegypti*, *Cx. quinquefasciatus* and *An. gambiae*. During DENV infection of *Ae. aegypti*, the genes in the Toll pathway (*GNBP*, *Toll5A* and *MYD88* genes) were upregulated in the salivary glands. Silencing of *MYD88*, caused a slight increase of DENV viral titre in the midgut [66]. Upon viral infection, Rel1 and its downstream antimicrobial peptides is upregulated to control infection against DENV [14, 75] and SINV [24], whereas in *Culex* mosquitoes, following WNV infection, the transcription factor Rel2 of the Imd pathway activates the secretion of an antiviral peptide against WNV infection [28].

### The Delta-Notch signaling pathway: a complementary pathway in regulating antiviral immunity

The evolutionarily conserved signaling pathway, Delta-Notch, plays crucial roles in embryonic development, stem cell maintenance and adult tissue renewal [76]. While the Delta-Notch signaling pathway was well described for its role in developmental processes, a recent study has reported a new role of the Delta-Notch signaling pathway in antiviral innate immunity in the mosquito, by limiting the replication of DENV in *Ae. aegypti* mosquitoes [77]. Notch is a transmembrane receptor and signaling depends on the binding of Delta ligands, which activates the proteolysis of the Notch receptor, releasing an active fragment, known as the Notch intracellular domain (NICD) that enters the nucleus to activate downstream target genes [76, 78]. During DENV infection, components of this pathway including *Delta*, *Notch* and *Hindsight* genes were also shown to be upregulated in *Ae. aegypti* mosquitoes [77]. Although the exact mechanism of how this signaling pathway limits DENV replication is not known, this study showed that activation of this signaling pathway induced endoreplication, in which cells undergo many rounds of DNA replication without mitosis to increase dramatically the genomic DNA content in the cells. Induction of endoreplication increased the number of gene transcripts that are involved in controlling viral spread [77].

## The cellular immune mechanisms in mosquitoes

The cellular defense response includes phagocytosis, nodulation and encapsulation of pathogens by hemocytes [20]. Furthermore, hemocytes also elicit humoral responses by activation of downstream signaling as previously mentioned and their effector responses lead to the synthesis and secretion of soluble effectors molecules such as AMPs and components of the phenoloxidase cascade into the hemolymph to control infection against invading pathogens [79].

### Hemocyte-mediated antiviral immunity in mosquitoes

Hemocytes are cells that circulate within hemolymph, and are permissive to viral infection including DENV [15], SINV [80] and WNV [81]. The hemocyte-mediated immune response is immediate and includes pattern recognition, phagocytosis, nodulation, melanization, production of antimicrobial peptides and initiation of signaling cascades for cytotoxic effectors to clear infection [20, 80, 82].

Hemocytes exist in two forms: circulating (circulate within hemolymph) and sessile (tissue resident). Furthermore, different populations of hemocytes have been described in mosquitoes. Studies have categorized mosquito hemocytes into prohemocytes, oenocytoids and granulocytes [83]. Granulocytes are the most abundant and constitute 80–95% of circulating hemocytes. Granulocytes are phagocytic, and upon activation they rapidly adhere to and engulf foreign particles [84]. Oenocytoids (~10% of circulating hemocytes) are the main producer of components of the phenoloxidase (PO) cascade in response to infection [85]. The PO cascade is a humoral immune response initiated by pathogen-associated pattern recognition molecules and leads to proteolytic processing of prophenoloxidase (PPO) to PO, which catalyzes the formation of melanin around invading pathogens [86]. The reaction intermediates generated from the proteolytic processes have been shown to inactivate SFV [86]. SFV has been shown to activate PO-based melanization cascade in mosquito cells, which results in inhibition of virus spread indicating that this pathway mediates the antiviral response in mosquitoes [86]. Nodulation occurs when multiple hemocytes bind to bacterial aggregates to form a multicellular sheath and the nodule formation is the main insect cellular defense reaction to clear a large number of bacteria from the hemolymph [20, 21].

### Fat body-mediated antiviral response of mosquitoes

The insect fat body is an organ that functions analogous to both adipocytes and livers in mammals. The fat body is crucial in regulating metabolism and growth in insects, and is responsible for energy storage, synthesis and secretion of hemolymph proteins and circulating metabolites [87]. A recent study reported that the JAK-STAT pathway is activated in the fat body of *Ae. aegypti* during dengue virus infection [68]. Overexpression of the *Dome* or *Hop* gene in the fat body of *Ae. aegypti*, resulted in inhibition of DENV infection in these transgenic mosquitoes, but this inhibitory effect was not observed for CHIKV and ZIKV, indicating that different viruses elicited the JAK-STAT pathway differently [68].

As the fat body is important in mediating antiviral responses in mosquitoes, its components such as cellular lipids may play a role as well. It has been shown that cellular lipids are manipulated by flaviviruses to facilitate viral replication. In *Ae. aegypti* cells, SINV and DENV infection resulted in accumulation of lipid droplets (LDs) [88]. LDs are made up of a monolayer of fatty acid and other structural proteins including Perilipin 1, 2 and 3. LDs are found in the fat body tissue of mosquitoes and their main function is maintaining lipid homeostasis, by regulating biogenesis and degradation of LDs [88]. LDs serve as a reservoir of lipids which are important for anchoring the viral replication machinery for efficient viral replication [89]. Exploitation of lipid metabolism has also been reported in WNV, indicating the importance of lipids in pathogenesis [90]. Genes involved in LD biogenesis and lipid metabolism are upregulated upon DENV infection [88]. Interestingly, activation of immune signaling pathway, including the Toll and the Imd pathways enhanced LD content in mosquito midgut [88]. During DENV infection, fatty acid synthase is recruited to the site of replication by DENV nonstructural protein 3 to stimulate fatty acids synthesis [91]. Furthermore, inhibition of fatty acid synthase decreased DENV viral titers and thus serve as a potential antiviral target to control viral infections [92].

### Autophagy to promote antiviral immunity

Autophagy is an evolutionarily conserved process that sequesters and mediates the degradation of cellular components, such as proteins and organelles, to maintain cellular and tissue homeostasis [93]. Autophagy involves the sequestration of damaged organelles or misfolded proteins by forming double-phospholipid membrane vesicles, known as autophagosomes. The autophagosomes then fuse with lysosomes to mediate the degradation of sequestered contents within the lysosome [93, 94]. In addition to the role of autophagy in maintaining cellular and tissue homeostasis, a protective role for autophagy against intracellular pathogens including viruses has been shown in mammalian systems and, to a lesser extent, in *Drosophila* [95–97]. In *Drosophila*, antiviral autophagy against vesicular stomatitis virus (VSV) and Rift Valley fever virus (RVFV) is activated through pathogen recognition by the Toll-7 transmembrane receptor. The activation of Toll-7 leads to the activation of autophagy *via* the phosphatidylinositol 3-kinase (PI3K)-Akt-signaling pathway, which is an autophagy pathway that senses the status of nutrient availability. Upon activation, autophagy is able to limit viral replication in flies. Furthermore, loss of Toll-7 leads to an increase in viral RNA production in *Drosophila* cell line [97] and *Toll-7* mutant flies which are more susceptible to RVFV infection [95, 96], suggesting that there is a role for autophagy in controlling viral replication. Due to the conservation of autophagy, it is postulated that the autophagy pathway is also involved during viral infection of mosquitoes. For example, during DENV infection, autophagy is activated to generate energy for viral replication. In particular, autophagy regulates lipid metabolism by degradation of the lipid droplets to release lipids that undergo oxidation to generate energy for viral replication [91, 98]. However, the role of autophagy during virus infection of mosquitoes is still largely unknown.

## Current mosquito control strategies

The prevention and control of mosquito-borne diseases is primarily reliant on vector control measures, such as the use of insecticides, mosquito nets and environmental management to limit human-vector contact [5]. Over the last decade, approaches such as the release of *Wolbachia*-infected mosquitoes [99, 100] and genetically modified mosquitoes [101, 102] into native mosquito populations have been undertaken. These approaches aim to either reduce viral capacity in vector populations or reduce reproductive success.

### *Wolbachia*-infected mosquitoes

*Wolbachia pipientis* are symbiotic bacteria, vertically transmitted from mother to offspring, and exist naturally in an estimated 60% of insects [103]. Recently, the *w*Mel strain of *Wolbachia* has been introduced into *Ae. aegypti*, which is not a natural host of *Wolbachia*, in an attempt to limit their ability to transmit important arboviruses including DENV, CHIKV and ZIKV. To date, ten countries, including Australia, Brazil and Vietnam, have participated in field trials for DENV control by releasing *Wolbachia-*infected mosquitoes into the wild [104]. In controlled field releases in Cairns, Australia, the *w*Mel strain of *Wolbachia* was successfully established in natural populations of *Ae. aegypti* mosquitoes [100]. Several years later, the *Wolbachia* infection rate in the mosquito population remains high [105]. Additionally, *Wolbachia-*infected mosquitoes from the same field populations continue to demonstrate reduced susceptibility to DENV under laboratory conditions [106]. Field and clinical studies in Vietnam showed that *w*Mel-infected *Ae. aegypti* are not permissive to DENV infection when the mosquitoes were fed with patient-derived viremic blood meals [107].

Despite the potential of *Wolbachia* as a useful and effective tool to combat mosquito-borne diseases, the mechanisms of how *Wolbachia* mediate viral replication in mosquitoes remains largely unclear. However, there are likely to be multiple mechanisms involved: (i) priming the immune system by inducing reactive oxygen species (ROS) and activating innate immune genes to secrete effector proteins such as Vago to limit viral replication [108, 109]; (ii) direct competition for cholesterol between viruses and *Wolbachia* [110]; and (iii) perturbations in vesicular trafficking, lipid metabolism, intracellular cholesterol trafficking and in the endoplasmic reticulum (ER) [111]. Despite promising results from field trials, many concerns need to be addressed before *Wolbachia*-infected mosquitoes can become a safe and effective strategy to suppress arbovirus transmission. For example, one study has reported that *Wolbachia-*infection of *Ae. aegypti* increased the infection rates of other insect-specific flaviviruses that are not medically important [112]. Secondly, *Wolbachia*-based mosquito control might not be effective for other mosquito species. For example, *Wolbachia*-infection of *Cx. tarsalis*, which is a novel WNV vector in North America, enhanced the infection rate of WNV [113]. Additionally, *Wolbachia*-infected *Anopheles* mosquitoes exhibited an enhanced susceptibility to *Plasmodium* infection, thus increased the risk of malaria transmission by these mosquitoes [114].

### CRISPR/Cas9 genetically-modified mosquitoes

The clustered regularly interspaced short palindromic repeats/CRISPR associated sequence 9 (CRISPR/Cas9) system has recently emerged as a powerful genome editing tool to combat vector-borne diseases [115, 116]. In addition, the use of the CRISPR/Cas9 system to genetically modify mosquitoes to combat mosquito-borne diseases is steadily growing. For example, the body of literature on the development of highly effective CRISPR/Cas9 systems has expanded significantly especially within the last five years since the emergence of this system. Briefly, the CRISPR/Cas9 system comprises two components: (i) a small RNA (17–20 bases) known as single guide RNA (sgRNA) that is designed to complement the target genomic DNA sequence; and (ii) *Streptococcus pyogenes* Cas9 nuclease (SpCas9) that binds and cleave the double-stranded target DNA in the presence of a short conserved sequence (2–6 nucleotides), known as protospacer-associated motif (PAM) [115]. The SpCas9 endonuclease complexes with the sgRNA and induces double-stranded DNA breaks at the target DNA sequence [115]. The PAM sequence for SpCas9 is NGG, and SpCas9 will not bind to the target DNA sequence if PAM is absent at the site. Interestingly, the frequency of NGG in the *Ae. aegypti* genome is relatively high (approximately once every 17 base pairs). This feature makes CRISPR/CAS9 an efficient and reliable system to make precise changes to the genome of this vector species [115, 117].

Recently, CRISPR/Cas9-based gene editing has been widely used as an efficient tool to modify the mosquito genome of *An. stephensi* [116], *Ae. aegypti* [115], *Cx. quinquefasciatus* [118] and *Cx. pipiens* [119]. More recently, CRISPR/Cas9 has been used to generate a knockout mutant of the fibrinogen-related protein 1 (*FREP1*) gene of *An. gambiae*, which encodes for a specific immune protein that is important for parasite’s midgut infection stage to block malaria transmission [101].

## Conclusions

Currently there are no suitable vaccines nor cure for the majority of mosquito-transmitted diseases. Vector control remains the gold standard strategy to block disease transmission. More recently, genetically-modified mosquitoes have been developed and field tests are ongoing, as potential alternative strategies to control disease transmission by mosquitoes. However, these strategies are not perfect and insufficient to block transmission. Furthermore, as these strategies are still novel, little is known about how viruses and mosquito defense mechanisms may evolve to reduce the efficacy of these strategies. More extensive knowledge of how mosquitoes respond to infection, how the innate immune system controls virus infection, other host factors that facilitate viral replication, how viruses persist in mosquitoes and how different mosquito species or strains vary in permissiveness to virus infection at the molecular level could improve and maximize the effectiveness of current strategies and could possibly result in identification of new molecular targets for new vector control strategies.

## Abbreviations

PRRs: pattern recognition receptors
PAMPs: pathogen-associated molecular patterns
YFV: yellow fever virus
DENV: dengue virus
JEV: Japanese encephalitis virus
WNV: West Nile virus
ZIKV: Zika virus
CHIKV: chikungunya virus
SINV: Sindbis virus
SFV: Semliki Forest virus
RRV: Ross River virus
ONNV: O’nyong-nyong virus
AMPs: antimicrobial peptides
ROS: reactive oxygen species
JAK-STAT: Janus kinase-signal transducer and activator of transcription
Imd: immune deficiency
RNAi: RNA interference
siRNAs: small interfering RNAs
miRNAs: microRNAs
pri-miRNA: primary microRNA
pre-miRNA: precursor microRNA
piRNA: Piwi-interacting RNA
dsRNA: double-stranded RNA
Dcr-2: Dicer-2
RISC: RNA-induced silencing complex
ssRNA: single-stranded RNA
Ago2: Argonaute-2
Piwi: P-element induced wimpy testis
Aub: Aubergine
Ago3: Argonaute 3
piRISC: piRNA-induced silencing complex
VSV: vesicular stomatitis virus
CRISPR/Cas9: clustered regularly interspaced short palindromic repeats/CRISPR associated sequence 9
sgRNA: single guide RNA
